# Case of Idiopathic Tetanus

**Published:** 1850-12

**Authors:** D. G. Gregory

**Affiliations:** La Grange, Texas


*Case of Idiopathic Tetanus.* By D. G. Gregory, M. D. of La Grange, Texas.

On Sunday, 15th Sept. 1850, I was called to see Miss Mary Kersens, aged 20, a native of Germany, who emigrated to Texas in the spring of 1850. Says she always enjoyed good health; commenced to menstruate at 13 years of age, and has menstruated regularly ever since. Finding herself here without friends or relatives, she went to live in the family of Mr. P. V. Shaw, of La Grange, Texas. She was taken sick about the first inst., (after a hard day's work, washing and scrubbing,) with a chill, followed by fever, accompanied by severe paroxysms of cramp. Several physicians of this place saw her, one of whom attended her for a number of days, and from some cause (unknown to me) abandoned the case. The above history I had from herself.

When I first saw her on the above day I found her presenting the following symptoms, viz.: soreness and tenderness on pressure of the whole spinal column, the least pressure upon the spinous processes producing the most exquisite pain; the most severe spasm, affecting the whole muscular system, and producing complete opisthotonos, trismus, and sometimes risus sardonicus; tongue slightly coated in the middle, clean at the edge; pulse and skin natural; paroxysms coming on suddenly, and of about thirty minute's duration; entire consciousness, and freedom from pain in the intervals. I commenced the treatment by applying a blister to the whole length of the vertebral column, gave quinine gr. v., morphiæ sulph. gr. ss. every two hours; enema of gruel and fourth proof brandy.

Monday, 16. Symptoms same as yesterday; treatment same, with the exception that I increased the quantity of quinine and morphine, and as the bowels had not been evacuated last night, ordered an enema of tartar emetic in solution, suspended in gruel, to be repeated in the afternoon. The blister having drawn well, a strong solution of sulphate of morphia was ordered to be applied to the blistered surface, and also to be repeated in the afternoon.

Tuesday, 17. Patient rather worse than yesterday. Slight operation on the bowels, paroxysms longer in duration and more severe, lasting nearly an hour, with emprosthotonos. Ordered Calomel, gr. x. S. Quiniæ gr. viii., Morphiæ Sulphat gr. ss., every two hours, and to be continued all day; enema at night of Ol. Ricini ℥i., suspended in gruel. At this time I sent for Dr. I. Evans, my partner, as consulting physician, who, with me, attended the case until dismissed, cured.

Wednesday, 18. Copious discharges from the bowels last night, slight degree of ptyalism, paroxysms not so severe as yesterday, but longer in duration, (one lasting for the space of an hour and a half) the intervals between paroxysms longer also. Continued same treatment as yesterday.

Thursday, 19. Patient better to-day, considerable ptyalism apparent, omitted Hydrargyri Sub. Mur., and continued Quinine and Morphia, with Prussiate Iron gr. ii. added to each dose.

Friday, 20. Patient improving, treatment as yesterday. Copious discharges of very offensive matter from the bowels, at intervals during the day and night.

Saturday, 21. Patient still improving, medicines discontinued, ptyalism receding, patient sat up a little in bed.

Sunday, 22. Forenoon, patient a little worse, in consequence of having exposed herself to a draft of cool air in an open gallery yesterday; resumed Quinine and Morphia and gave it every three hours.

Six o'clock, P. M., was called in haste to see our patient; on arriving found her much worse than on any previous visit, the paroxysms being very severe, attended with the most excruciating pain, extorting from her the most lamentable shrieks of agony; the body and face distorted into every conceivable attitude, presenting, alternately, opisthotonos, emprosthotonos, pleurothotonos, risus sardonicus, and trismus; her condition at this time was truly pitiable, and none who saw her supposed she could survive more than a few hours. Ordered Calomel gr. viii., Mor. sulph. gr. i., Quinine gr. viii., every two hours, to be continued all night.

Monday, 23. No perceptible amendment in the symptoms, ptyalism again increasing, bowels evacuated last night; continued same treatment as yesterday; administered enema of Ol. Ricini ℥i., Ol. Turpentine ʒss., applied a large blister over the left side.

Tuesday, 24. More complete ptyalism, patient completely narcotized. Oil and turpentine evacuated the bowels, symptoms more favorable, discontinued the Calomel; Quinine and Morphia as before.

Wednesday, 25. Patient a great deal better; cramp confined pretty much to the lower extremities; continue Quinine and Morphia every four hours.

Thursday, 26. Still improving very fast, no spasm to day; patient sat up nearly all day.

Friday, 27, to Sunday, 29. Patient still went on improving, until Sunday evening, when she had slight paroxysms, which were effectually relieved by the application of a blister on the side, a few doses of Quinine, and two doses of Seidlitz powders, during the night.

Monday, 30, to Oct. 15. Patient has still gone on improving, and expresses herself as well as ever she was.

*Remarks.*—I consider the above case one of idiopathic tetanus of miasmatic origin. The most remarkable feature in the symptoms, was the excruciating pain attending the paroxysms, and their always commencing in the left side; and that most remarkable in the treatment is the enormous quantity of calomel and quinine taken before ptyalism was induced, and the circumstance that whenever it was, the symptoms were alleviated.

*La Grange, Texas, Oct.* 15, 1850.

